# Extracellular vesicles fail to trigger the generation of new cardiomyocytes in chronically infarcted hearts

**DOI:** 10.7150/thno.62304

**Published:** 2021-11-02

**Authors:** Bruna Lima Correa, Nadia El Harane, Manon Desgres, Maria Perotto, Paul Alayrac, Chloé Guillas, Laetitia Pidial, Valérie Bellamy, Emilie Baron, Gwennhael Autret, Keirththana Kamaleswaran, Chloé Pezzana, Marie-Cécile Perier, José Vilar, Antonio Alberdi, Alain Brisson, Nisa Renault, Massimiliano Gnecchi, Jean-Sébastien Silvestre, Philippe Menasché

**Affiliations:** 1 Université de Paris, PARCC, Inserm, F-75015 Paris, France.; 2 University of Geneva, Translational Research Centre in Onco-hematology, Department of Medicine, Geneva, Switzerland.; 3 Department of Molecular Medicine, Unit of Cardiology, University of Pavia, Pavia, Italy and Laboratory of Experimental Cardiology for Cell and Molecular Therapy, Fondazione IRCCS Policlinico San Matteo, Pavia, Italy.; 4 Department of Cardiology, Hôpital Européen Georges Pompidou, F-75015 Paris, France.; 5 Inserm, UMRS-976, Hôpital Saint-Louis, Paris, France.; 6 UMR-CBMN, Université de Bordeaux, CNRS, IPB, F-33600 Pessac, France.; 7 Fujifilm Cellular Dynamics, Inc, Madison, Wisconsin, USA.; 8 Department of Cardiovascular Surgery, Hôpital Européen Georges Pompidou, F-75015 Paris, France.

## Abstract

**Background:** Extracellular vesicles (EV) mediate the therapeutic effects of stem cells but it is unclear whether this involves cardiac regeneration mediated by endogenous cardiomyocyte proliferation.

**Methods:** Bi-transgenic MerCreMer/ZEG (n = 15/group) and Mosaic Analysis With Double Markers (MADM; n = 6/group) mouse models underwent permanent coronary artery ligation and received, 3 weeks later, 10 billion EV (from human iPS-derived cardiovascular progenitor cells [CPC]), or saline, injected percutaneously under echo guidance in the peri-infarcted myocardium. Endogenous cardiomyocyte proliferation was tracked by EdU labeling and biphoton microscopy. Other end points, including cardiac function (echocardiography and MRI), histology and transcriptomics were blindly assessed 4-6 weeks after injections.

**Results:** There was no proliferation of cardiomyocytes in either transgenic mouse strains. Nevertheless, EV improved cardiac function in both models. In MerCreMer/ZEG mice, LVEF increased by 18.3 ± 0.2% between baseline and the end-study time point in EV-treated hearts which contrasted with a decrease by 2.3 ± 0.2% in the PBS group; MADM mice featured a similar pattern as intra-myocardial administration of EV improved LVEF by 13.3 ± 0.16% from baseline whereas it decreased by 14.4 ± 0.16% in the control PBS-injected group. This functional improvement was confirmed by MRI and associated with a reduction in infarct size, the decreased expression of several pro-fibrotic genes and an overexpression of the anti-fibrotic miRNA 133-a1 compared to controls. Experiments with an anti-miR133-a demonstrated that the cardio-reparative effects of EV were partly abrogated.

**Conclusions:** EV-CPC do not trigger cardiomyocyte proliferation but still improve cardiac function by other mechanisms which may include the regulation of fibrosis.

## Introduction

Except for cardiac transplantation, the current treatments of ischemic heart failure do not address the root cause of the disease, *i.e.*, the loss of a large number of cardiomyocytes and this observation has been a strong incentive for transplanting therapeutic stem cells with the hope of replacing tissue loss by new functional cardiac cells. However, the parallel and consistent observation that few, if any, of these cells engrafted permanently has led to challenge the exogenous cell-based “remuscularization” hypothesis and led to consider an alternate mechanism of action based on the paracrine harnessing of endogenous pathways contributing to cardiac repair 1. Further mechanistic insights have then led to recognize that these paracrine effects were largely mediated by biomolecules packaged in extracellular vesicles (EV). These nanoparticles harbour a biologically rich cargo made of proteins, lipids and nucleic acids that they shuttle into recipient cells, thereby modulating their function. The most compelling evidence for the role of EV as mediators of the stem cells effects have come from the findings that they could recapitulate the cardio-protective potential of their parent cells in pre-clinical models of multiple diseases including ischemic heart injury 2-5. In a clinical perspective, EV-based therapies are currently raising a growing interest because of their attractive features including a large-scale production more akin to that of a pharma-type product, their lack of immunogenicity (depending of the parental cells) 6 and an off-the-shelf availability due to the possibility of storing them at minus 80 °C without loss of bioactivity 7.

However, the precise mechanism(s) whereby EV improve cardiac function remain elusive. Since one of them could be the generation of new cardiomyocytes, the present study was designed to assess, with the use of two transgenic models, whether EV could trigger endogenous cardiomyocyte proliferation in the setting of chronic heart failure 2.

## Material and Methods

### Overview of experimental protocols

**Figure 1:**
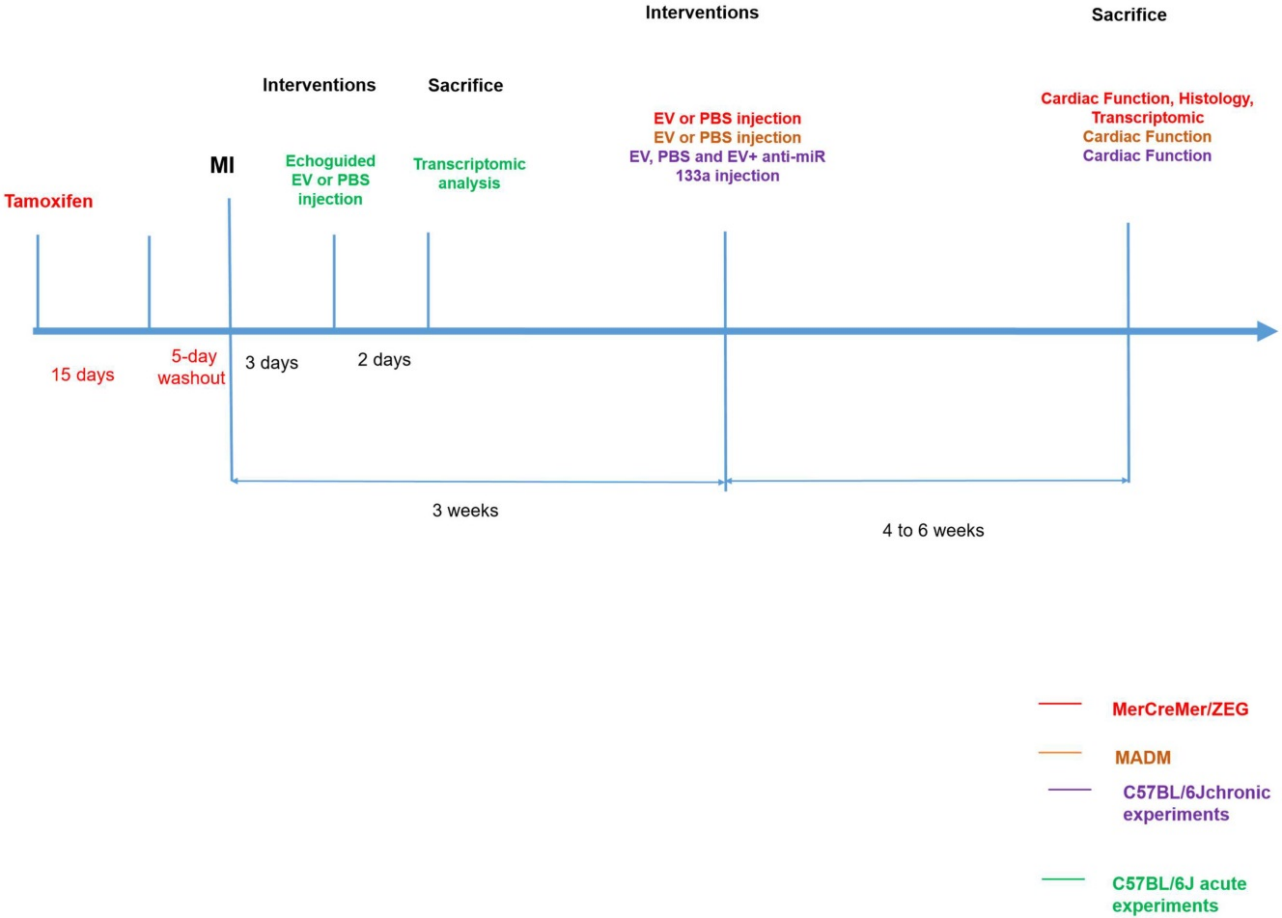
Experimental protocols.

Extracellular vesicles were collected from cardiovascular progenitor cells (CPC) differentiated from human induced pluripotent stem cells. A myocardial infarction was created in mice by a permanent left coronary artery ligation followed, three weeks later, by transcutaneous injections, under echocardiographic guidance, of EV or Phosphate-Buffered Saline (PBS) in the peri-infarction area. This approach was chosen to avoid a repeat surgery which is fraught with a high mortality rate in previously infarcted mice 8. Three consecutive series of experiments were performed. We first used the transgenic MerCreMer/ZEG model to assess whether new cardiomyocytes had been generated and identify their putative source (division of existing cardiomyocytes or mobilization of another tissue-resident cell source). In this model, an injection of tamoxifen induces existing cardiomyocytes to express the Green Fluorescent Protein (GFP) and thus to stain green. Osmotic minipumps delivering 5-ethynyl-2´-deoxyuridine (EdU) were also subcutaneously implanted for 7-10 days at the time of PBS or EV injections to enable the subsequent tracking of double-labeled cells for GFP and EdU used as a marker for cardiomyocyte proliferation. To double-check the results yielded by this first series, we then proceeded with the transgenic Mosaic Analysis with Double Markers (MADM) model in which cardiomyocytes, if they divide, produce daughter cells that are either red or green whereas if they fail to divide, they remain double-colored (yellow) or colorless. The ratio of single-colored to double-colored cells thus indicates whether cytokinesis, a hallmark of cell division, has occurred 9. In a mechanistic perspective, we finally used C57BL/6J mice to more specifically assess the role of a microRNA (miR-133-a1) overexpressed in the EV-treated MerCreMer/ZEG hearts through the use of one of its antagonists. In all series, mice were sacrificed at 4-6 weeks after injections. Outcomes were assessed on echocardiography, magnetic resonance imaging (MRI), immunohistochemistry and gene expression in the myocardium. The different experimental protocols are illustrated in Figure 1 and all procedures are detailed in Supplemental Materials.

### Statistical Analysis

Apart from genome-wide analyses, Mann-Whitney or Student t-Test were used. Analyses were conducted using GraphPad InStat3 (8.0.1) and performed by an independent statistician blinded to the treatment groups. Values are given as mean ± standard error (SEM) and a *p* value < 0.05 was considered statistically significant. Mice that died within 24 h of coronary artery ligation surgery (< 10% in all genotypes studied) were excluded from subsequent analyses.

## Results

### EV-CPC quantification

The majority of EV (~ 70%) were in the size range of small EV approximately 50 to 200 nm (Supplemental Figure 1A). The concentration of particles of the size of small EV in the media prior to cell exposure was 4x10^8^ particles/mL as measured by Nanoparticle Tracking Analysis. This value represented the background noise which was taken into account when the actual number of EV released by the CPC (higher by two logs) was measured. The identity of EV-CPC was confirmed by their positive expression of phosphatidylserine, CD9, CD63 and HSC70 (Supplemental Figure 1B-D) while they were unstained for calnexin (Figure S1E).

### EV-CPC do not trigger endogenous cardiomyocyte proliferation but still improve cardiac function

**Figure 2:**
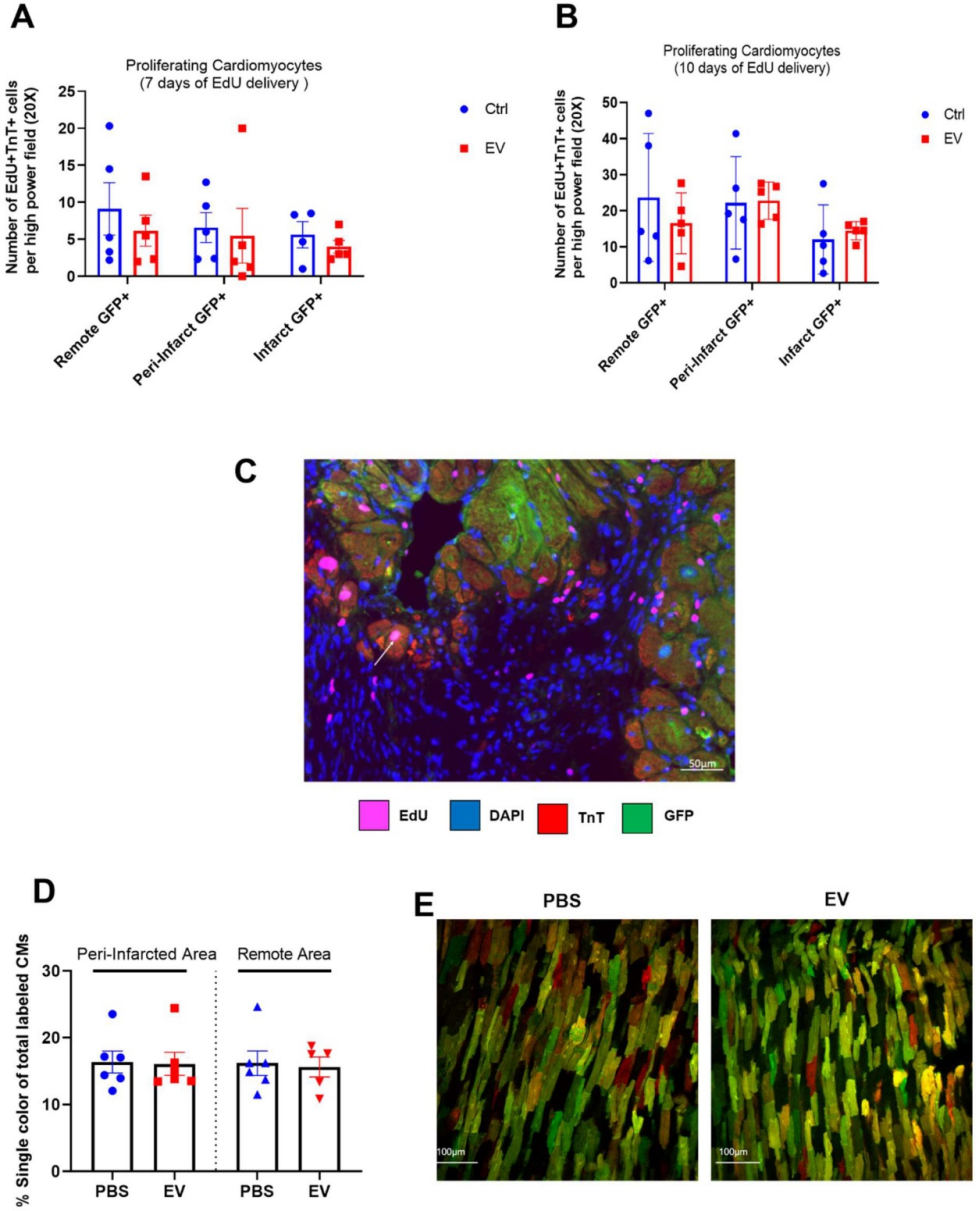
** Absence of EV-induced cardiomyocyte proliferation in murine adult hearts.** Mean number of proliferating cardiomyocytes (EdU+ and cTnT+) after 7 (A) or 10 (B) days of EdU delivery. Each group consisted of 5-6 randomly selected mice. C. Representative fluorescence microscopic imaging of triple immune-staining for cTnT, GFP and EdU in MerCreMer/ZEG mice. Arrow points to a proliferating cardiomyocyte. D. Cardiomyocyte proliferation in MADM mice expressed as the percentage of single-coloured cardiomyocytes relative to the total number of cardiomyocytes per high-power field and showing similar results in the PBS-injected and EV-CPC-treated groups (n = 5-6). E. Representative fluorescence of bi-photon imaging.

In MerCreMer/ZEG mice, a 4-OH-tamoxifen pulse induced GFP expression only in cardiomyocytes, with 80% of them expressing the fluorescent protein. The number of proliferating (EdU^+^) cardiomyocytes (TnT^+^) did not significantly differ between groups at either 7 or 10 days of EdU delivery (Figure 2A-B). These values represented approximately 0.6% and 1.7% of the total cardiomyocyte content per tissue section. Importantly, all proliferating double-positive EdU^+^ TnT^+^ cardiomyocytes stained positive for GFP (Figure 2C), thereby demonstrating that cardiac proliferation derived from pre-existing cardiomyocytes.

**Figure 3:**
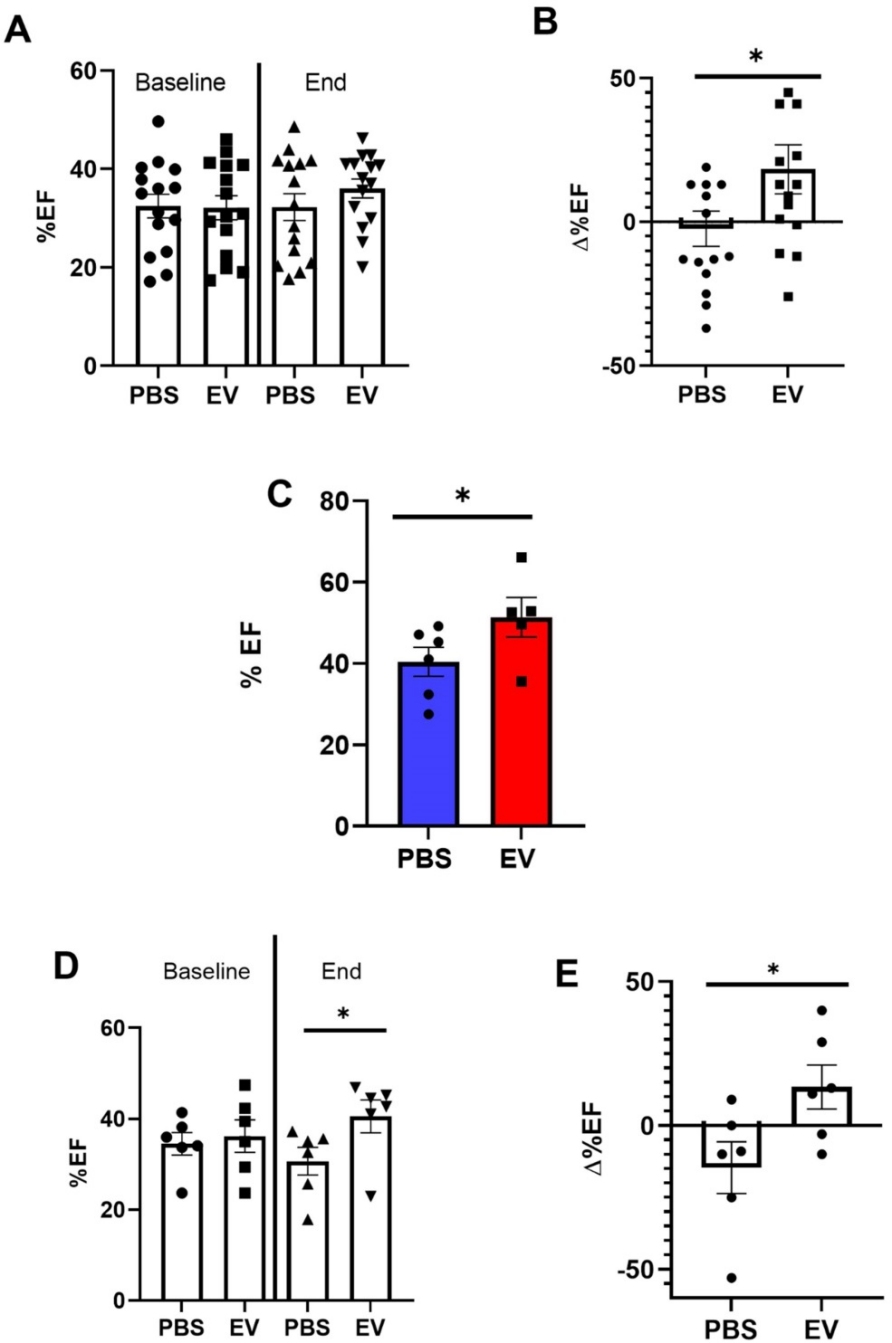
** Effects of EV-CPC on left ventricular function.** A. Absolute echocardiographic values of left ventricular ejection fraction expressed at baseline (pre-transplantation, 3 weeks following MI) and at the end of study or B. As percent changes from baseline in MerCreMer/ZEG mice. Each group comprised 15 mice. *p ≤ 0.05. C. Absolute values of left ventricular ejection fraction, as measured by Magnetic Resonance Imaging, in MerCreMer/ZEG mice. There were 5 and 6 randomly selected mice in the EV and PBS groups, respectively. *p ≤ 0.05. D. Echocardiographic data of left ventricular ejection fraction expressed at baseline (pre-transplantation, 3 weeks following MI) and at the end of study or E. As percent changes from baseline in MADM mice. Each group comprised 6 mice. *p ≤ 0.05

However, while left ventricular ejection fraction (LVEF) was not different between the two groups 3 weeks after myocardial infarction (referenced as baseline, Figure 3A), their patterns of changes then diverged since at the end-study time point, LVEF had increased by 18.3% ± 0.2% (from baseline) in the EV-treated hearts which contrasted with a decrease by 2.3% ± 0.2% in the PBS group (Figure 3B). This was associated with a decrease of left ventricular end-diastolic (LVEDV) and end-systolic volumes (LVESV) by 3 ± 0.2% and 4 ± 0.3%, respectively, in the EV-treated group which contrasted with an increase by 20 ± 0.2% and 24 ± 0.2%, respectively, in the control group (Supplemental Figure 2A-B). This improvement of LVEF following EV injections was confirmed by MRI which was performed in a subset of mice randomly selected from the two groups (Figure 3C).

MADM mice featured a similar pattern as intra-myocardial administration of EV improved LVEF by 13.3 ± 0.16% from baseline whereas it decreased by 14.4 ± 0.16% in the control PBS-injected group (Figure 3D-E). However, in keeping with the data obtained with the MerCreMer/ZEG model, we failed to show any difference in the number of single-colored cells between the two groups, thereby indicating a similar rate of cardiomyocyte proliferation independent of the EV treatment (Figure 2D-E). Taken together, these results suggest that EV administration did not promote a substantial proliferation of pre-existing cardiomyocytes in the failing heart.

### EV-CPC decrease infarct size and fibrosis

**Figure 4:**
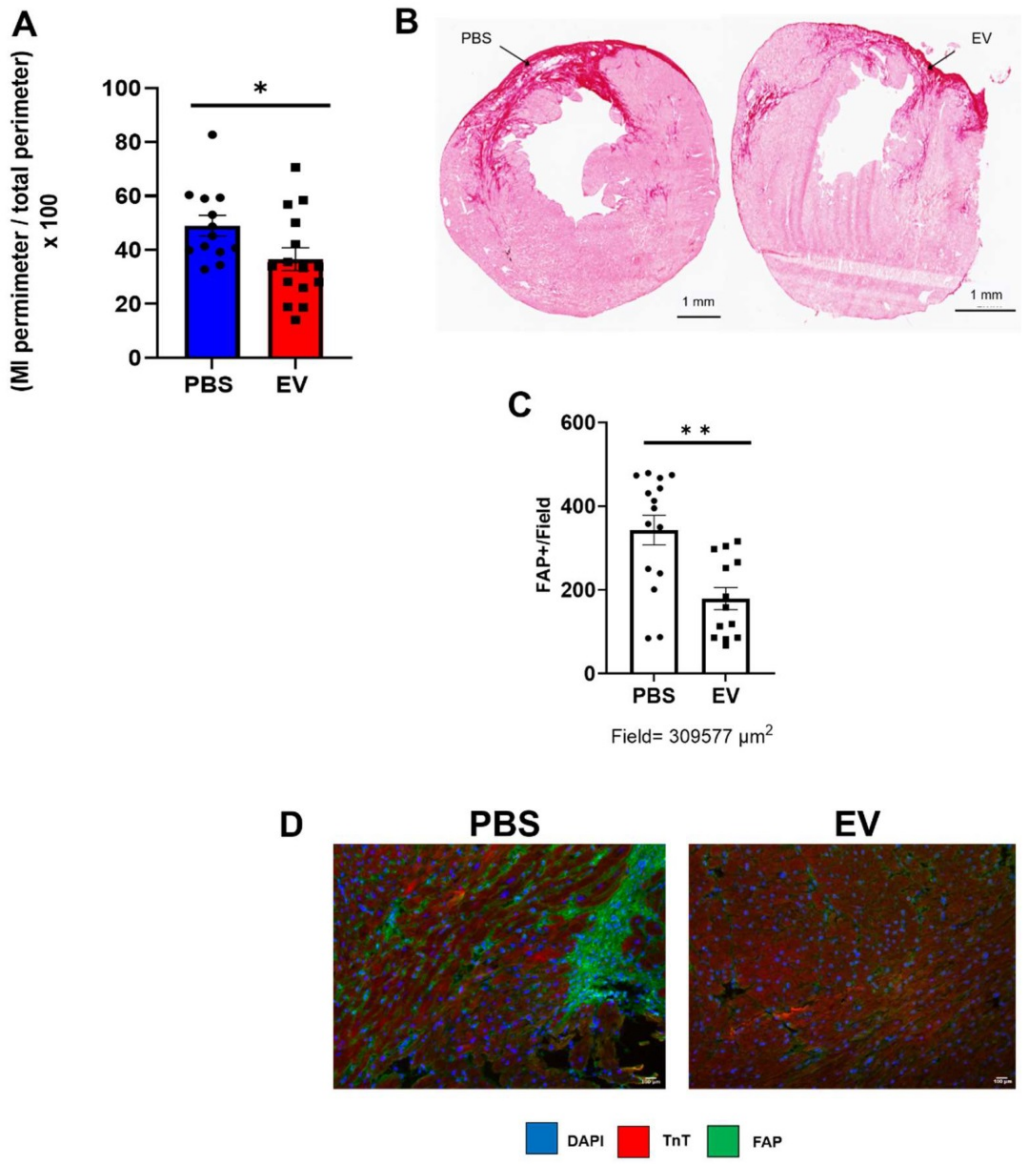
** Effect on EV-CPC on fibrosis in MerCreMerZEG mice.** A. Infarct size expressed as a ratio between the perimeter of the infarct and the total perimeter of the section (H&E staining). For this end point, there were 15 and 13 mice in the EV and PBS groups, respectively. B. Representative images of the infarct area in a control and an EV-treated heart (Sirius red staining). Arrows point to infarcted area. C. Quantification of Fibroblast Activated Protein (FAP) in peri-infarcted area in EV-CPC (n = 13) and PBS (n = 15) injected hearts. D. Representative immunostaining for FAP (green) and cardiac Troponin T (red) in the left ventricular free wall section of a PBS-injected control heart and a EV-injected heart. (20X). *p ≤ 0.05 **p ≤ 0.005.

Conversely, administration of EV reduced myocardial fibrosis. MerCreMer/ZEG mice injected with PBS had a mean infarct size, assessed by Hematoxylin and Eosin staining, of 49 ± 10.7% versus 36.6 ± 12.7% in those that had received EV, yielding a significant (p = 0.02) difference of 12.4 ± 5.7% (Figure 4A-B). The reduction in infarct size and interstitial fibrosis in EV-treated hearts was confirmed by Masson trichrome and Sirius red staining, respectively (Figure S3A-B). This was paralleled by a decreased (albeit not significant) expression of several pro-fibrotic mRNAs (Col 1a1, Col 3a1, Lox, MMP3, Supplemental Figures 3C) and a significant decrease in the number of Fibroblast Activated Protein (FAP)-positive cells in EV-treated hearts compared to controls (Figure 4C-D). Likewise, in the C57BL/6J mice in which transcriptomics studies were performed acutely (*i.e.*, 5 days post MI, 2 days post intervention) pro-fibrotic genes were also down-regulated, the difference between EV-treated and PBS controls being significant for MMP3 (p = 0.0352) and elastin (p = 0.0058) (Supplemental Figure 4). Capillary density and cardiomyocyte hypertrophy did not differ between groups (data not shown).

**Figure 5:**
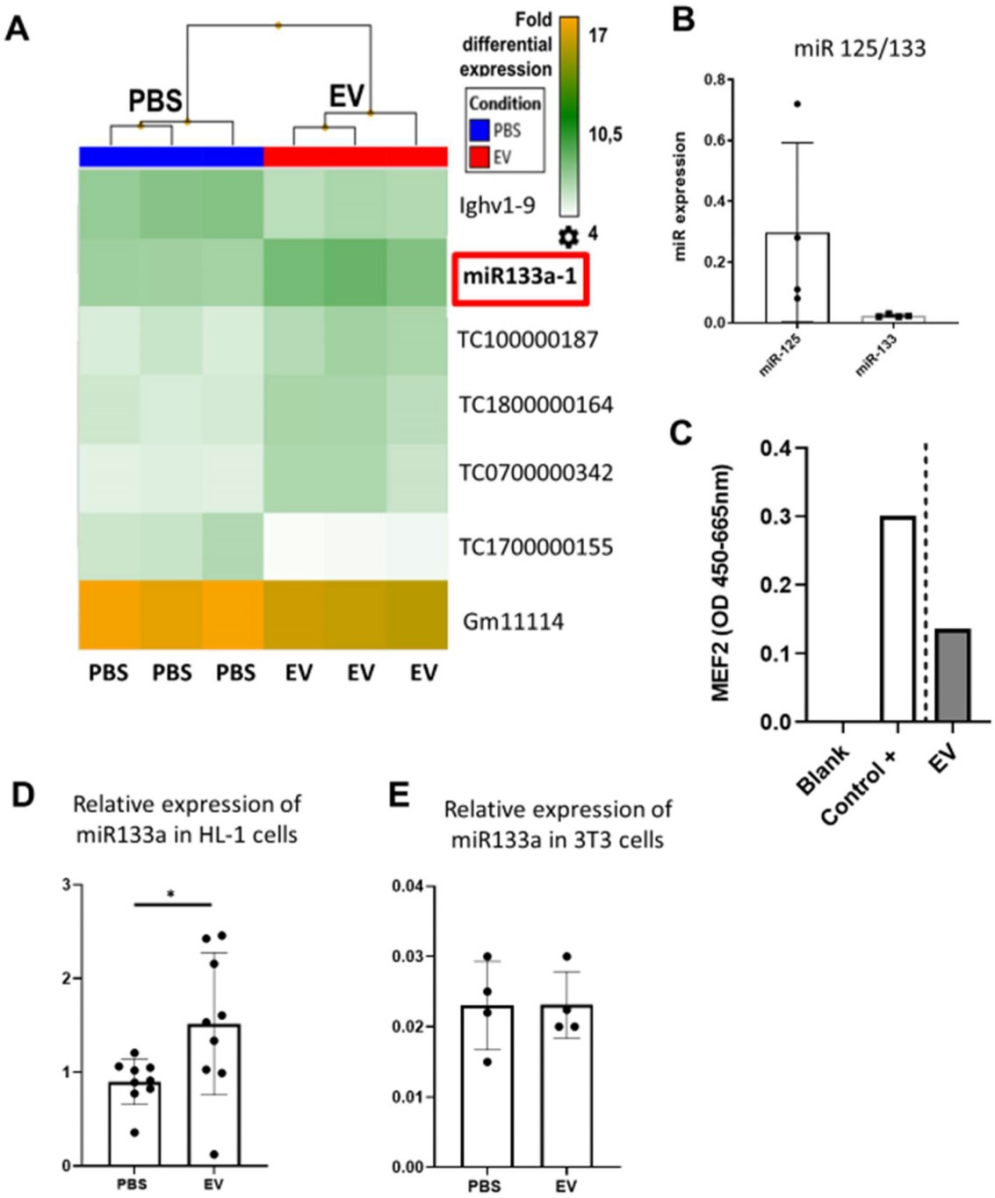
** Effects of EV-CPC on transcriptomics in MerCreMerZEG mice.** A. Hierarchical clustering of the Whole Transcript (WT) Expression Arrays was performed using mRNAs from control and treated hearts and differentially expressed genes (DEG) were selected using one-way analysis of variance (ANOVA). The fold change (FC) of every gene, together with their corresponding p-value, were used for selection of DEGs with a cut-off of 2.0. (n = 3/group). B. Expression levels of miR-133 in EV-CPC. miR125 was taken as the reference. C. Expression of MEF2 in EV-CPC. The control was nuclear extracts provided by the manufacturer (2 technical replicates). D. Expression levels of miR-133a in HL-1 cells treated with PBS or EV after incubation for 24 h. E. Expression levels of miR-133a in 3T3 cells treated with PBS or EV after incubation for 24 h.

**Figure 6:**
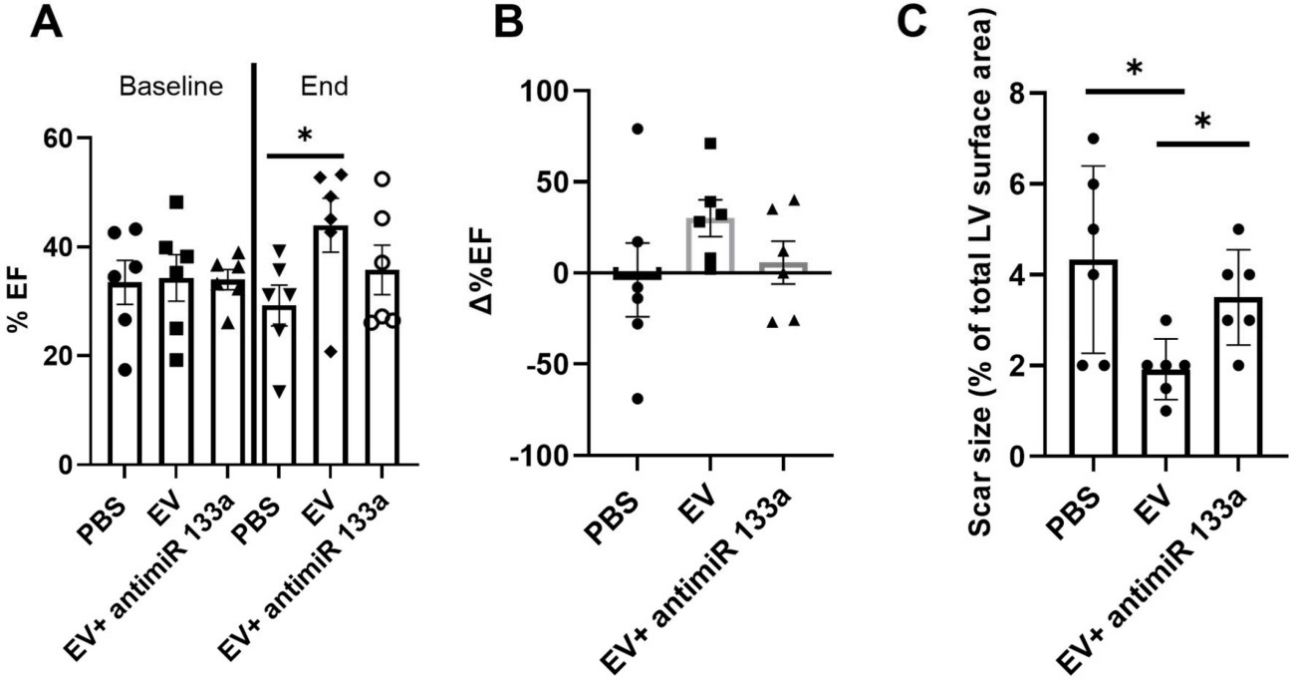
** Effects of EV-CPC on left ventricular function and fibrosis in C57BL/6J mice.** A. Echocardiographic data of absolute values of left ventricular ejection fraction expressed at baseline (pre-transplantation, 3 weeks following MI) and at the end of study in C57BL/6J mice or B. As percent changes from baseline, showing a partial abrogation of the protective effects of EV-CPC in the presence of a miR133 antagomir. Each group comprised 6 mice. C. Infarct size expressed as a ratio between the infarct area and the total area of the section (Masson's trichrome staining). *p ≤ 0.05.

To provide additional insight into the mechanism(s) of EV-induced cardiac repair, genome-wide microarrays were used to assess the differential expression of RNAs in the myocardial tissue of EV-CPC-treated MerCreMer/ZEG mice compared with PBS-injected animals (Figure 5A). Out of the 64,000 gene arrays, 14 genes were differentially expressed, of which 9 have been studied in mammalians (Table 1). In line with the histological findings, the anti-fibrotic miR-133-a1 10-12 and its pseudo-gene Gm21747 were found significantly upregulated compared with controls (by 2.13 and 2.94 fold, respectively, Table 1). However, only small amounts of miR-133-a1 were found in EV-CPC (Figure 5B), making unlikely that its increased expression in the EV-treated hearts was due to an EV-mediated direct transfer. As an alternate possibility was that EV foster an endogenous overexpression of miR-133-a1 in targeted cardiac resident cells, we assessed the expression, in the EV cargo, of the transcription Myocyte enhancer factor-2 (MEF2), which is a miR-133-a1 activator,^13^ and actually found it increased (Figure 5C). Additional *in vitro* experiments confirmed the upregulation of miR-133-a1 in cardiomyocytes (Figure 5D), but not in fibroblasts (Figure 5E)**,** when these cell types were incubated in the presence of EV. In an attempt to further assess the potential relationship between the increased expression of miR-133-a1 and the EV-induced improvement in heart function, we conducted an additional series of *in vivo* experiments where C57BL/6J mice were injected with EV-CPC, PBS or a mix of EV-CPC and anti-miR133. In keeping with the previous functional data, EV-CPC improved significantly LVEF by 14.76% ± 6.20% compared to PBS controls 4-6 weeks after treatment whereas this protective effect was partly abrogated by the anti-miR133 antagomir (Figure 6A-B). Likewise, the fibrosis-reducing effect of EVs was partly lost following their co-delivery with the anti-miR-133 antagomir (Figure 6C). Left ventricular volume changes did not differ between groups (Figure S5).

## Discussion

There is now compelling evidence that the primary mechanism of action of intramyocardially transplanted cells is an activation of endogenous repair pathways 1. This paracrine effect is largely mediated by the EVs released by the grafted cells owing to the unique ability of these nanoparticles to shuttle a biologically rich cargo into recipient cells and thus modulate several signaling pathways involved in the stimulation of angiogenesis and the mitigation of inflammation, apoptosis and fibrosis 6, 14-16. However, less is still known about the potential capacity of EVs to trigger cardiomyocyte proliferation. The present study was designed to address this issue.

The main results of our present work can be summarized as follows: (1) EV fail to induce a substantial proliferation of host cardiomyocytes, (2) they nevertheless preserve cardiac function of chronically infarcted hearts and (3) this benefit seems rather mediated by a regulation of myocardial fibrosis.

Several reports have already shown that EV released by CPC, either native^4^ or differentiated from pluripotent stem cells 3, 14 improve function of chronically infarcted hearts. The present study confirms a benefit of EV on LVEF across three different mouse strains and with the use of two imaging modalities (echocardiography and MRI).

To track the generation of new endogenous cardiomyocytes, we used two transgenic models but failed to demonstrate a substantial EV-induced cardiomyocyte proliferation with either one. In the MerCreMer/ZEG experiments, a triple immunostaining allowed to label host cardiomyocytes expressing cTnT (constitutively), GFP (following the tamoxifen pulse chase) and EdU (for cells which had proliferated) but there was no difference in the number of these triple-positive cardiomyocytes between the control and EV groups. To confirm these findings, we also used the MADM model which provides unambiguous evidence for cell division directly related to cytokinesis 17,18 and while it is recognized that cell division is methodologically challenging to document directly, this model system is considered as one of the most sensitive 9. In our experiments, the ratio of single-colored cells to double-colored cells was identical in the two groups, thereby ruling out again an effect of EVs on cell proliferation. Although one could argue that newly generated cardiomyocytes might have originated not from the already mature ones but from endogenous progenitors, this is unlikely since such cells would have been positive for both cTnT and EdU but negative for GFP, which did not actually happen. While the mechanism(s) underlying the exit of cardiomyocytes from cell cycle shortly after birth in mammals are still debated, a likely contributor to this arrest is the switch from anaerobic glycolysis to oxidative phosphorylation 19. To our knowledge, there are no data supporting that EV could reverse this switch and restore an energetic milieu fostering cardiomyocyte proliferation. At the opposite, they have been reported to shuttle mitochondria as part of the package they transfer into recipient cells 20,21 and, as such, could rather favor mitochondria-based oxidative metabolism. Altogether, our results are in line with the concept that division of cardiomyocytes stops shortly after birth (or only persists at a very low rate 22) and the finding that EV from CPC exert a cardio-protective effect without triggering a cardiomyocyte proliferation 23.

Nevertheless, the lack of *de novo* cardiomyocyte proliferation did not preclude an improvement in heart function which was related, at least in part, to a mitigation of fibrosis demonstrated histologically and by the trend for a downregulation of fibrosis-inducing genes in the myocardial tissue. The effects of EV on the mitigation of fibrosis have been documented in previous studies 16 and are consistent with their ability to modulate ischemia-induced myocardial inflammation 6. Of note, in the present study, there was a significantly decreased immune-histochemical expression of Fibroblast Activated Protein (FAP) whose inactivation by chimeric antigen receptor T-lymphocytes has recently been shown to contribute to improve heart function 24. In line with this hypothesis, there was an increased expression of the anti-fibrotic miR-133-a1 in the EV-treated hearts 10-12. While our data do not show a direct link between the EV components, the myocardial upregulation of miR-133 and the reduction of fibrosis, several lines of evidence jointly support such a relationship*:* (1) the low content of miR-133-a1 in EV makes unlikely that its increased expression in EV-treated hearts resulted from its direct EV-mediated horizontal transfer; (2) in contrast, the EV cargo was enriched in MEF-2, which has been shown to activate transcription of a RNA encoding miR-133a-1 13. This supports the hypothesis of a miR133-a myocardial upregulation of endogenous origin, triggered by other EV components than EV-enclosed miRNA 25 and our data show that EV can upregulate the expression of miR-133a in cardiomyocytes and not fibroblasts; (3) one of the targets of miR-133a is ColA1A, a main collagen fiber present in the heart. Of note, in a rat angiotensin II-dependent hypertension model, an increased content of ColA1A in interstitial and perivascular fibrosis is associated with a down-regulation of miR-133a 26. In keeping with these observations, we evidenced that EV treatment reduced heart tissue levels of Col1A1; (4) the co-delivery of EV with an antagomir of miR-133a resulted in functional outcomes intermediary between those of control PBS-injected and EV-treated hearts with a partial abrogation of the EV-associated anti-fibrotic effects. Put together, these results strongly suggest the likely contribution of miR-133-a1 to the EV-associated cardiac repair; at the same time, the antagomir experiments where abrogation of the EV protective effects were only partially mitigated highlight that these effects cannot be reduced to a single compound and rather result from the cooperative action of the multiple constituents of their cargo. The practical implication is that deconstructing the EV cargo may not be the most effective approach for leveraging its reparative capacities, supporting our choice to rather target the EV-enriched secretome for our clinical trial under preparation.

We acknowledge that our data are at variance with those reported in other studies. However, these discrepancies could be explained by important differences in the design of the experiments, including (1) the usual delivery of EV at the time of injury 27-29 (and not at the more chronic stage, like in our protocol) with expected differences in local cardio-instructive cues, (2) the use of cells of a different origin (the epicardium) 29, cultured under hypoxic conditions^30^ or genetically engineered 27, and (3) the use of outcome measures failing to provide direct evidence for cytokinesis 30. Of note, in the study of Balbi et al. 28 where exosomes secreted by CPC from human atrial explants induced cardiomyocyte proliferation by a periostin-dependent mechanism, the exosome-induced improvement in left ventricular function was unchanged after periostin inhibition, thereby suggesting a minimal, if any, contribution of the exosome-induced cardiomyocyte cell cycling in the preservation of cardiac function.

Some limitations of this study should finally be outlined. Direct intramyocardial injections of EV likely leads to their rapid wash-out from the myocardium and thus may fail to provide enough exposure time for them to exert therapeutically relevant effects; however, the observation of EV-associated benefits other than cardiomyocyte proliferation, *i.e.*, mitigation of fibrosis and improvement of function, makes this assumption questionable even if, in the future, incorporation of EV in slow-release hydrogels could be an efficacious means of extending their exposure to the myocardium and consequently of potentiating their effects 31. Along this line, and for sake of minimizing the number of confounding factors, we used a single-dose intramyocardial mode of delivery but cannot exclude that repeated dosing made possible by a less invasive approach, like the intravenous route, could not result in different outcomes. Second, functional data were collected 4-6 weeks after EV injections and therefore do not allow to determine whether the observed improvement is, or not, sustained over time. Finally, we did not test the effects of a miR-133-a1 mimics to double-check the role of this miRNA in fibrosis mitigation.

Despite these shortcomings, the results of this study strongly suggest that EV from CPC have cardio-protective effects which, at least at the dosing which was used, do not seem to involve the generation of new cardiomyocytes but are more likely mediated by a regulation of myocardial fibrosis. Other strategies currently aim at inducing such a cardiac "regeneration" by lifting checkpoint inhibitors (like the Hippo pathway) 32, overexpressing cell-cycle regulators 18 or converting host fibroblasts into cardiomyocytes 33 but their clinical feasibility, efficiency and safety still remain to be established. In the meantime, the use of EV could yet be a more user-friendly and effective addition to the current armamentarium of therapies against heart failure.

## Supplementary Material

Supplementary methods and figures.

## Figures and Tables

**Table 1:**
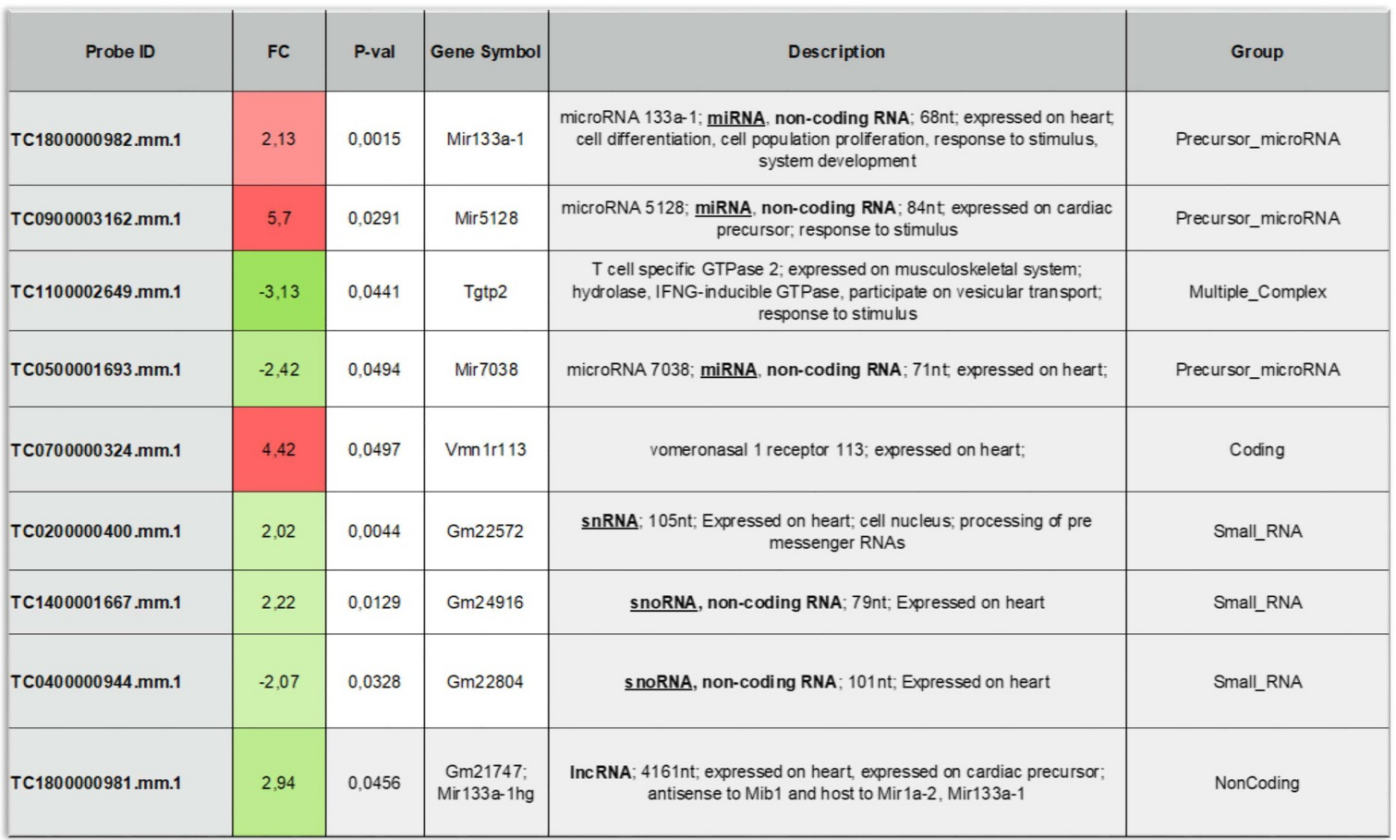
List of the differentially expressed genes between the EV-CPC- and PBS- groups (n = 3/group).

## Abbreviations

EV: Extracellular vesicles
CPC: cardiovascular progenitor cells
PBS: phosphate-buffered saline
GFP: Green Fluorescent Protein
EdU: 5-ethynyl-2´-deoxyuridine
MADM: Mosaic Analysis with Double Markers
MRI: Magnetic resonance imaging
NTA: Nanoparticle Tracking Analysis
TnT: Troponin T
LVEF: Left ventricular ejection fraction
LVEDV: left ventricular end-diastolic volume
LVESV: left ventricular end-systolic volume
Col: Collagen
Lox: Lysyl oxidase
MMP3: Matrix Metalloproteinase
FAP: Fibroblast Activated Protein
RNA: Ribonucleic acid
MEF-2: Myocyte enhancer factor-2
EV-CPC: Extracellular vesicles from iPS-derived cardiovascular progenitor cells
b-FGF: basic fibroblast growth factor
BCA: bicinchoninic Acid Assay
MISEV: Minimal Information for Studies of Extracellular Vesicles
NP: nanoparticles
EM: electron microscopy
HRP: horse radish peroxidase
ECL: enhanced chemiluminescence
Tg: Transgene
PCR: Polymerase Chain Reaction
RT-PCR: Real-Time Quantitative Reverse Transcription Polymerase Chain Reaction
OCT: Optimal Cutting Temperature
RT: Room temperature
BSA: bovine serum albumin
FITC: Fluorescein isothiocyanate
DAPI: 4', 6-diamidino-2-phenylindole
WGA: Lectin Wheat Germ Agglutinin
hmbs: hydroxymethylbilane synthase
GADPH: glyceraldehyde 3-phosphate dehydrogenase
