# Supplementary material for: Extracellular vesicles fail to trigger the generation of new cardiomyocytes in chronically infarcted hearts
Source: Theranostics. 2021 Nov 2;11(20):10114–24. doi: 10.7150/thno.62304 (PMC8581432; doi:10.7150/thno.62304)
Supplement: Supplementary file 1 — Supplementary methods and figures. [file thnov11p10114s1.pdf]

Supplemental Materials

Extracellular Vesicles Fail to Trigger the Generation of New Cardiomyocytes in Chronically Infarcted Hearts

Bruna Lima Correa*[1], Nadia El Harane*[2], Manon Desgres[1], Maria Perotto[3], Paul Alayrac[1], Chloé Guillas[1], Laetitia Pidial[1], Valérie Bellamy[1], Emilie Baron[4], Gwennhael Autret[1], Keirththana Kamaleswaran[1], Chloé Pezzana[1], Marie-Cécile Perier[1], José Vilar[1], Antonio Alberdi[5], Alain Brisson[6], Nisa Renault[7], Massimiliano Gnecchi[3], Jean-Sébastien Silvestre[1], Philippe Menasché[1,8].

Short Title: Extracellular Vesicles for Heart Failure

Affiliations

1. Université de Paris, PARCC, Inserm, F-75015 Paris, France
2. University of Geneva, Translational Research Centre in Onco-hematology, Department of Medicine, Geneva, Switzerland
3. Department of Molecular Medicine, Unit of Cardiology, University of Pavia, Pavia, Italy and Laboratory of Experimental Cardiology for Cell and Molecular Therapy, Fondazione IRCCS Policlinico San Matteo, Pavia, Italy.
4. Department of Cardiology, Hôpital Européen Georges Pompidou, F-75015 Paris, France.
5. Inserm, UMRS-976, Hôpital Saint-Louis, Paris, France.
6. UMR-CBMN, Université de Bordeaux, CNRS, IPB, F-33600 Pessac, France.
7. Fujifilm Cellular Dynamics, Inc, Madison, Wisconsin, USA.

8. Department of Cardiovascular Surgery, Hôpital Européen Georges Pompidou, F-75015 Paris, France.

Ethics section

All procedures were approved by the Institutional Ethics Committee from Paris University (project #04073.01) and complied with European legislation (European Commission Directive 2010/63/EU) on animal care. All animals received anesthesia by isoflurane (1.5 L/min isoflurane at 0.8-1.0 L/min of O_2) and were euthanized by an overdose of isoflurane (3.5 L/min isoflurane at 2.0 L/min of O_2).

Cell Culture, Extracellular Vesicle Preparation and Characterization

Cell Culture and EV isolation

Cryopreserved cardiovascular progenitor cells differentiated from human induced pluripotent stem cells (iPSC-CPC) (CPC-301-020-001-PT, iCell®, Cellular Dynamics International, Madison, WI) were thawed and plated at a density of 78,000 cells/cm^2 on fibronectin-coated flasks (Roche Applied Sciences, St. Quentin Fallavier, France) and cultured for 4 days with 7% CO_2 at 37 °C in serum-free media (William's E medium) with Cocktail B from hepatocyte maintenance supplement pack, 25 µg/mL Gentamicin (Life Technologies, Illkirch, France) and bFGF, 1 µg/mL (Miltenyi, Bergisch Gladbach, Germany). During iPSC-CPC culture, the media was refreshed on day 2 and on day 4, the conditioned media containing the extracellular vesicles (EV) secreted over the previous 48 h was collected for EV isolation. Conditioned media was then pre-cleared by centrifugation for 6 min at 1200 *g* and EVs were pelleted by ultracentrifugation for 16 h at 100,000 *g*. We have previously shown that these cells feature a phenotype consistent with their progenitor developmental stage evidenced by the reduced expression of the pluripotency markers (NANOG, SOX2, OCT3/4, LIN28), the expression of

early cardiovascular lineage markers (ISL-1, MEF2C, GATA4, and NKX2.5) and the absence of mature cardiomyocyte markers [1].

EV quantification and characterization

Extracellular vesicles were quantified by Nanoparticle Tracking Analysis ([NTA] NanoSight LM-14, Malvern, Palaiseau, France), and protein content was measured by a Bicinchoninic Acid Assay ([BCA] (Thermo Scientific, Illkirch, France) as previously described [1,2]. The EV-CPC were characterized following the Minimal Information for Studies of Extracellular Vesicles guidelines[4], using evaluation of CD9, CD81, CD63 expression by Western Blot, immuno-gold labeling cryo-transmission electron microscopy and flow cytometry as previously described [2,3]. Each batch of EV-CPC was tested *in vitro* using scratch-induced endothelial cell wound healing and H9c2 cardiomyoblasts viability assays, as previously described [2]. Another series of experiments were added to further characterize EV-CPC according to MISEV guidelines [3].

Immuno-gold labeling and cryo-transmission electron microscopy

Gold nanoparticles (NPs) (10-nm diameter) were conjugated with an anti-CD81 monoclonal antibody (mAb) (clone JS64, Beckman Coulter) or with annexin-A5 (Anx5) following procedures previously described [4]. EV samples were labeled for 1 h with anti-CD81- or with Anx5-gold-NP at 1-3 × 10^{15} gold-NP/L, at ambient temperature. Immuno-gold labeled samples were processed for cryo-EM as follows. A 4-µL aliquot was deposited on an EM grid coated with a perforated carbon film. After draining the excess liquid with a filter paper, grids were quickly plunged into liquid ethane using a Leica EMCPC cryo-chamber and stored in cryo-boxes under liquid nitrogen until EM observation. For cryo-EM observation, grids were mounted onto a Gatan 626 cryoholder and transferred to a Tecnai F20 microscope (FEI, USA) operated at 200 kV. Images were recorded with an Eagle 2k CCD camera (Thermo Fisher, USA).

Western Blot

CD63 and HSC70

After thaw, EV-CPC aliquots were concentrated again with ultrafiltration 0.5 (Merck Millipore, PMNL 30Kda, Ref UFC 5030). The retentate was divided into two parts: one was directly quantified by NTA and denatured in loading buffer (4X Laemmli Sample Buffer without β-mercapto-ethanol, Bio Rad, Ref 1610747) at 95 °C for 5 min, the second was lysed with Ripa buffer (Thermo-Scientific, Ref 89900), protein titrated (Micro BCA™ Protein Assay Kit assay, ThermoFischer, Ref 232325) and then denatured in loading buffer without β-mercapto-ethanol at 95 °C for 5 min. Approximatively 1.10^9 particles or 20 µg of lysate proteins were loaded on 1-D polyacrylamide gels (4-20%) (Mini protean TGX precast, Bio-Rad Ref 4561094) and submitted to electrophoresis in running buffer (10X Tris-Glycine-Sodium dodecyl sulfate [SDS], Bio-Rad, Ref 1610732) for 2 h at 20V then at 60V for 2 additional hours in a Mini-PROTEAN Tetra Vertical Electrophoresis Cell (Bio Rad). Nitrocellulose membranes (Precut, Bio-Rad, Ref 1620146) were pre-wetted in water and transfer buffer before use. Proteins were then transferred onto the nitrocellulose membranes in a wet Mini Trans-Blot Cell (Bio-Rad) with blotting buffer (10X Tris-Glycine, Bio-Rad, Ref 1610734, supplemented by 20% ethanol, pH fixed at 8.3 to improve the resolution of small molecular weight proteins) at 125V for 1 h. Membranes were then blocked in 3%-milk-TBS-T blocking buffer (10X Tris Buffered Saline, Bio-Rad, Ref 1610735, supplemented by 0.01% Tween 20 and 3% skimmed milk) in constant shaking for 1 h. CD63 was stained with purified mouse anti-human clone H5C6 (BD Pharmingen, Ref 556019, diluted 1/1500) and HSC70 was stained with HSC70/HSP73 rat monoclonal antibody (clone 1B5, Enzo Life Sciences, ref ADI-SPA-815, diluted 1/1500) overnight in blocking buffer with constant shaking. Membranes were then cut to separate the incubation with HRP-conjugated goat anti-mouse secondary antibody (ImmunoReagents Inc, Ref GtxMu-003-DHRPX, diluted 1/1500) from the incubation with HRP-conjugated secondary

Peroxidase AffiniPure Donkey Anti-Rat IgG (Jackson laboratory, ref 712-035-153, diluted 1/1000) in blocking buffer with constant shaking for 1.5 h. After primary and secondary antibodies incubations, blots were washed three times with TBS-T during 15 min and two times during 5 min. Revelation was performed with enhanced chemiluminescence (ECL) substrate (ThermoScientific, Ref 34075) and the chemiluminescent signal was revealed using the ImageQuant™ LAS 4000.

CD9

Protein samples were denatured in Laemmli buffer for 5 min at 95 °C (BioRad, 1610792) and separated based on molecular weight by electrophoresis in 4-12% gradient gels (BioRad Criterion™, 3450123) for 1h30 at 110 V. Proteins were then transferred on 0.45 µm nitrocellulose membranes (BioRad, 1620094) during 20 min at 110 V, following which the membranes were incubated with Ponceau Red to verify the efficiency of the transfer process. Membranes were blocked with 5% (w/v) milk in TBS supplemented with 0.1% Tween-20. To detect the protein of interest, membranes were then incubated with primary antibodies (Anti-human CD9, Merck Millipore CBL162) overnight at 4 °C, with constant agitation. After three 10-minute washes, membranes were incubated with a secondary anti-mouse antibody coupled with HRP (Amersham, GE Healthcare, 1/3000) for 1 h at room temperature. Immunodetection was performed using Clarity™ Western ECL Substrate, and the chemiluminescent signal was revealed using the ImageQuant™ LAS 4000.

Calnexin

200.000 CPC were lysed with Ripa buffer (Thermo-Scientific, Ref 89900). The lysate of cells and the equivalent amount of EV-CPC secreted by 200.000 CPC (i.e. approximatively 1.10^9 particles) were then denatured in loading Laemmli Sample Buffer (Tris HCl pH 6.8 250 mM, SDS 280 mM, Glycerol 40%, Bromophenol Blue) at 95 °C for 5 min and loaded on 1-D 9% polyacrylamide gels (0.75 mm thin) and submitted to electrophoresis in running buffer (Tris

125 mM, Glycine 960 mM, SDS 17 mM) overnight at constant 0.05 mA in the electrophoresis chamber. Nitrocellulose membranes (Precut, Bio-Rad, Ref 1620146) were pre-wetted in water and transfer buffer before use. Proteins were then transferred onto the nitrocellulose membranes in a wet Mini Trans-Blot Cell (Bio-Rad) with blotting buffer (Tris 250 mM, Glycine 960 mM, 20% ethanol, 10% SDS) for 1h30 at constant 0.3 mA.

Membranes were then blocked in 3%-milk-TBS-T blocking buffer (Tris HCl pH 8,6 50 mM, NaCl 750 mM, 0.05% Tween 20 and 3% skimmed milk) in constant shaking for 1 h. Calnexin was then stained with rabbit polyclonal antibody (Enzo, Ref ADI-SPA-865, diluted 1/1000) overnight in blocking buffer with constant shaking and then incubated with HRP-conjugated goat anti-rabbit secondary antibody (Jackson ImmunoResearch, Ref AB_2338953 diluted 1/2500) in blocking buffer with constant shaking for 1 h. After primary and secondary antibodies incubations, blots were washed three times with TBS-T during 15 min and two times during 5 min. Revelation was performed with ECL substrate (ThermoScientific, Ref 34075) and the chemiluminescent signal was revealed using the ImageQuant™ LAS 4000.

Dose of EV-CPC

The doses used are expressed using different metrics according to MISEV guidelines for *in vivo* experiments. The EV-CPC secreted by an equivalent amount of 1.4×10^6 of CPC correspond to 1×10^{10} (+/- 3000) particles, as quantified by NTA and 100 µg of proteins, as measured by BCA (ThermoScientific).

Generation of the MerCreMer/ZEG mouse model

MerCreMer/ZEG mice harbor a tamoxifen inducible Cre-recombinase under the genetic control of the cardiomyocyte-specific alpha myosin-heavy chain promoter (MerCreMer transgene) and a ubiquitously expressed floxed LacZ gene plus stop codon upstream of a GFP locus [5] (ZEG transgene). These mice were obtained by breeding a MerCreMer female mouse (B6.Cg-Tg

(ACTB-Bgeo/GFP) 21Lbe/J) with a ZEG male mouse (Tg(CAG-Bgeo/GFP)21Lbe/J) which were kindly provided by Professor Richard Lee, Harvard University. For genotyping, tail tips were sampled, deoxyribonucleic acid (DNA) extracted, and polymerase chain reaction (PCR) was performed to identify double transgenic animals with primers listed below:

MerCreMer forward: 5′-GTCTGAC TAGGTGTCCTTCT-3′;

MerCreMer backward: 5′-CGTCCTCCTGCTGGTA TAG-3′;

ZEG forward: 5′-AAGTTCATCTGCACCACCG-3′;

ZEG backward: 5′-TCCTTGAAGAAGATGGTGCG-3′;

ZEG control forward: 5′-CTAGGCCA CAGAATTGAAAGATCT-3′;

ZEG control backward: 5′-GTAGGTG GAAATTCTAGCATCATCC-3′.

Double heterozygous MerCreMer-ZEG mice were used for the subsequent experiments.

Generation of the mosaic analysis with double markers (MADM) mouse model

Mosaic analysis with double markers mice with a high lox-induced efficiency upon recombination (MADM-ML) were obtained from the Jackson laboratory (JAX, Bar Harbor, Maine): Igs2tm1(ACTB-EGFP,-tdTomato)Zng/Igs2tm2(ACTB-tdTomato,-EGFP)Zng/J. To allow a cardiac-specific labelling, we crossed MADM-ML mice with animals expressing a constitutive cardiac-specific alpha myosin-heavy chain–Cre obtained from JAX laboratory: B6.FVB-Tg(Myh6-cre)2182Mds/J. In this model, cardiomyocytes that successfully divide in the presence of a cell-type-specific Cre-recombinase produce daughter cells that are either red, green, red and green (yellow) or colorless, based on allelic recombination of fluorescent reporters; however, if the cardiomyocytes fail to divide, they remain double-colored (yellow) if recombination occurs or colorless if there is no recombination. The identification of these animals was performed by PCR on DNA extracted from tail tips with primers listed below.

The identification of these animals was performed by PCR on extract DNA from tail tips with the following primers:

MADM-ML F1: 5'-AACTGGGCATGTGGAGACAGAGAA-3';

MADM-ML F2: 5'-GACCGCCGTCCTCGAAGTTCATCA-3';

MADM-ML F3: 5'GTTGCCGTCGTCCTTGAAGAAGAT-3';

MADM-ML Wild-type forward: 5'AATCCTTCAGCTGCCCACTCTACT-3';

MADM-ML Wild-type backward: CCCCAGGCATTTCTGCTTTTTCTGT-3'.

Myh6-Cre specific: 5' AGAAGCCTAGCCCACACCAGAAAT-3';

Myh6-Cre common: 5'AGAAGCCTAGCCCACACCAGAAAT-3';

Myh6-Cre endogenous: 5'CCCTGGTGCACCTGCTTGGGTTTC-3'.

Experimental model of chronic heart failure

Myocardial infarction (MI) was induced in MerCreMer/ZEG, MADM and C57BL/6J mice by permanent ligation of the left coronary artery. Two different protocols of infarction were implemented which differed by the timing of the treatment: in MerCreMer/ZEG and MADM mice, a chronic protocol was used with treatment given 3 weeks after the infarction and sacrifice 4-6 weeks thereafter. In the MerCreMerZEG series of mice, Cre recombinase expression was induced by a tamoxifen-containing diet (1-2 mg/pellet; 14 mg total; Envigo, Gannat, France) given for 14 days, followed by a 5-day wash-out before induction of infarction. In the C57BL/6J model, one part of the animals underwent an acute protocol with treatment implemented 3 days after infarction and sacrifice 2 days later for tissue collection and transcriptomic analysis; the remainder of mice went through the chronic protocol with sacrifice 4-6 weeks after treatment. Regardless of the timing, EV or a control Phosphate-Buffered Saline (PBS) solution were injected trans-cutaneously to avoid a second surgical procedure and its attendant mortality. To this end, mice were anesthetized with 2.5% isoflurane and immobilized on the VisualSonics

(Amsterdam, The Netherlands) platform, as previously described [6].Three injections of 15 µL each were performed under echocardiographic guidance in the anterior wall of the left ventricle (LV) in the peri-infarction zone, two in long axis view and one in lateral view. Extracellular vesicles were delivered at a dose of $1x10^{10}$ (± 3000) particles quantified by NTA, secreted by 1.4 million iPSC-CPC corresponding to 100 µg of proteins (BCA). In a separate set of experiments, 20 µM of the anti-microRNA (anti-miR) 133-a (Ambion by Life Technologies) were also co-delivered with EV. An equivalent volume of 45 µL was delivered in all PBS-injected control hearts. The different protocols are summarized in Figure 1.

Echocardiography

Three weeks after MI, all mice underwent a two-dimensional echocardiography (2 VEVO 2100, Visualsonics [Amsterdam, The Netherlands]). Images were acquired in long axis view in B-mode (VEVO Lab) and all parameters were calculated according to a previously described protocol [7]. All animals with a left ventricular ejection fraction (LVEF) > 45% were excluded. They were then assigned to the control and experimental groups to ensure that baseline values of LVEF and left ventricular enddiastolic (LVEDV) and endsystolic (LVESV) were well balanced between groups, thereby minimizing biases in the subsequent assessment of treatment effects. Four to six weeks after treatment, cardiac echography was performed again under the same conditions by the same operator blinded to the treatment groups.

Magnetic Resonance Imaging (MRI)

To validate echocardiographic results with a more operator-independent technique, 11 mice in the MerCreMer/ZEG series (EV n = 5, PBS n = 6) underwent MRI imaging in a Bruker BioSpec (Bruker, Wissembourg, France) adapted for small animal studies with a 4.7 Tesla magnetic field. Images were acquired for 85 s each using a FLASH-cine IntraGate technology and 1.03

mm-thick slices were oriented along the heart's short axis. Image analysis was carried out using the Cvi42 software (Circle Cardiovascular Imaging Inc. Calgary, Canada) for Windows 7 by two blinded operators.

5-ethynyl-2′-deoxyuridine (EdU) protocol

Osmotic minipumps (Alzet, Cupertino, CA) delivering EdU (EdU-Click 647 cell proliferation assay, Baseclick, Neuried, Germany) at 1 µg per hour per gram body weight were subcutaneously implanted for 7-10 days in MerCreMerZEG mice. Mice were sacrificed at 4-6 weeks following pump implantation and hearts and liver were collected.

HL-1 cells and 3T3 cells culture

Murine cardiomyocyte cells (HL-1 cells generously provided by Pr. Claycomb, LSU Health, School of Medicine, New Orleans, LA) or NIH/3T3 fibroblasts (ATCC® CRL-1658™, ATCC, Molsheim, France) were seeded at 250,000 cells per well and cultured until confluence in a 6-well plate. When confluence was reached, culture media was replaced with serum-deprived media and cells were treated with either EV-CPC ($10x10^9$ particles per well) or 0.1 µm filtered PBS for 24 h. Cells were then washed with filtered PBS and subsequently lysed with 800 µl QIAzol reagent (Qiagen, Courtaboeuf, France). Sample lysates were transferred into new tubes for miRNA extraction and quantification of miR 133-a by qRT-PCR.

Myocyte enhancer factor-2 (MEF-2) detection

Nuclear extraction of EV-CPC was performed using the Nuclear Extraction Kit (Abcam) and the extracts were quantified using BCA (ThermoScientific) for protein content. High throughput ELISA assay was used to quantify the MEF2 transcription factor (Abcam, Cambridge, UK) in EV-CPC.

Immunohistochemistry and immunofluorescence microscopy

After sacrifice, explanted hearts were washed in PBS, submerged in Optimal Cutting Temperature compound (OCT, Tissue-Tek; Sakura Finetek, Villeneuve-d'Ascq, France), frozen in liquid nitrogen and stored at -80 °C until sectioning. Hearts were cryo-sectioned into 5-10 µm thin-sections and an average of 12 tissue sections of different ventricular locations per heart were used for staining. To stain slides for Green Fluorescent Protein (GFP), cardiac Troponin T (TnT) and Fibroblast Activated Protein (FAP), fixation was performed with 4% paraformaldehyde for 15 min at room temperature (RT). Membrane permeabilization was achieved with 1% triton X-100 in PBS for 20 min at RT and non-specific epitopes were blocked with 5% bovine serum albumin (BSA) for 30 min at RT.

Triple GFP/TnT/ EDU staining

In the MerCreMer/ZEG mouse model, it was first necessary to check that after administration of Tamoxifen, mice expressed GFP exclusively in cardiomyocytes. A double staining for GFP and cardiac TnT was then performed with the relevant antibodies (FITC-conjugated anti-GFP, unconjugated anti-Troponin T and Texas red-conjugated secondary anti-rabbit antibody, all from Abcam). EdU was revealed according to the manufacturer's instruction by a click chemical reaction which yields a fluorescent dye (Cy5 Azide alternative). Nuclei were stained with 4', 6-diamidino-2-phenylindole (DAPI, Calbiochem, Lyon, France).

Double FAP/ TnT staining

Slides stained for FAP and TnT underwent a similar protocol using unconjugated anti-FAP (Fisher Scientific, Illkirch, France) and anti-TnT (Abcam) antibodies and Alexa 488-conjugated anti-rabbit (Life Technologies) and Alexa 594-conjugated anti-mouse secondary antibodies, respectively (Life Technologies). Nuclei were stained with DAPI.

Lectin Wheat Germ Agglutinin (WGA) and Isolectin staining

Slides stained for WGA and Isolectin underwent a similar protocol except that they were fixed for 10 min in acetone. Isolectin B4 and WGA were coupled to FITC and tetramethylrhodamine, respectively.

Hematoxylin/eosin staining

Thawed tissue sections were fixed in acetone for 10 min, washed 5 min in PBS and immersed for 2 s in hematoxylin and eosin (Sigma, Lyon, France). All slides stained were scanned with a slide scanner (NanoZoomer, Hamamatsu, Massy, France) and analyzed through the NDP view 2.5 software or the Metamorph® software (Molecular Devices, San Jose, CA) for fibrotic area calculation. Infarct size was calculated as the perimeter of the stained infarct area divided by the total perimeter of the section and expressed as a percentage.

Sirius Red staining

The Sirius Red staining was used to visualize and quantify fibrosis (area or interstitial). Picrosirius Red stains collagen fibers type I and III in red. All other cellular and extra-cellular elements acquire a variable yellowish to pinkish unspecific coloration. Tissues were fixed in 3.7% formaldehyde for 10 min and then immersed in Picrosirius Red for 16 min. Dehydrated was achieved through three subsequent baths each of 100% ethanol and 100% xylene. Slides were then mounted with Eukitt.

Masson Trichrome staining

The Masson Trichrome staining was used to visualize and quantify the scar area*,* Tissues were fixed in 3.7% formaldehyde for 10 min, washed twice (5 min), then immersed for 10 min in hematoxylin, washed three times (3 min), immersed in Culvert Fuchsin for 5 min, and washed again twice for 3 min, immersed this time in 1% of phosphomolybdic acid for 7 min, colored in light green for 5 min, and immersed in 1% of acetic acid for 3 s. Dehydrated was achieved through two subsequent baths each of 80% and 100% ethanol and three subsequent baths each of xylene. Slides were then mounted with Eukitt.

Image analysis

Images were analyzed by Qcapture (QImaging, Surrey, Canada), observed using a Leica DM 2000 optical microscope and quantified manually using the Metamorph® or Image J softwares.

Ribonucleic Acid (RNA) expression on mice hearts or murine cultivated cells

RNA extraction

RNAs were extracted from heart tissue cryo-sections (200-400 μm) or cultured cardiomyocytes and fibroblasts following the manufacturer's instructions and the concentration and purity of samples were evaluated with the NanoDrop® Spectrophotometer (ThermoFisher Scientific, Illkirch, France).

Affymetrix microarray analysis

RNA quantification and quality control were performed using the HT Standard RNA LabChip Kit and the Caliper LabChip Microfluidics System (Perkin Elmer, Villebon sur Yvette, France). One-hundred nanograms of total RNA were amplified, labeled, and fragmented using GeneChip WT. Each sample was hybridized onto Mouse Clariom D, washed, and stained with the Affymetrix® Fluidics Station 450. Array scanning was performed with the Affymetrix® GeneChip Scanner 3000 7G using the Command Console software (ThermoFisher Scientific, Illkirch, France) and then analyzed using the Affymetrix® rma-sketch routine. Subsequently, differentially expressed genes (DEGs) were selected using one-way analysis of variance (ANOVA) using the Affymetrix Transcriptome Analysis Console (TAC) software v.1.0. The fold change (FC) ($2 \geq -2$) of every gene, together with their corresponding p value (0.05) were used for selection of DEGs.

Real-time quantitative PCR (qRT-PCR)

qRT-PCR was used to assess the expression of genes involved in fibrosis, apoptosis, autophagy, proliferation and miR-133-a1 expression. The list of primers is indicated below. The primers

were designed using Primer Blasy with an annealing temperature of 50-60 °C. First-strand cDNA was synthetized from 250 ng of total mRNA with random hexamer primers using the RevertAid first-strand cDNA synthesis kit (Thermo Scientific). Quantitative PCR was carried out in 384-well plates on cDNA products diluted to 1/25 in duplicate using SYBR Green on a LightCycler® 480 (Roche) with the following parameters: initial denaturation step (95 °C, 5 min), then 40 cycles composed of denaturation (95 °C, 30s) and annealing/extension steps (60 C°-50 °C for 15s, 72 °C for 15s). Hmbs or GADPH were used as the housekeeping genes. The ratio between the amount of a target gene and that of the endogenous reference was then determined.

Col 1a1:

(F 5'ATTCCCGTTCGAGTACGGAA 3'; R 3'CTCGATCTCGTTGGATCCCT 5'),

Col 3a1:

(F 5'AAAGAGGATCTGAGGGCTCG 3'; R 5'TCACCTCCAACTCCAGCAAT3'),

Col 1a2:

(F 5' CGATGGCTGCTCCAAAAAGA 3'; R 5' AATGTCAAGGAATGGCAGGC 3'),

Lox:

(F 5' TGAACAAATAGCGGAGGGGC 3'; R 5' GAAAGCGCACAGAGTCTGGA 3'),

Bcl-2:

(F 5'TCTGGTTGGGATTCCTACGG 3'; R 5' AGGAGGGTTTCCAGATTGGG 3')

Elastin:

(F 5' CAGCTAAATACGGTGCTGCTG 3'; R 5'AATCCGAAGCCAGGTCTTG 3')

MMP3:

(F5'CTTCTGCAACTCCGACATCGT 3'; R 5' GGGGCATCTTACTGAAGCCTC3')

P53:

(F5'CTCACTCCAGCTACCTGAAGA 3'; R 5'AGAGGCAGTCAGTCAGTCTGAGTCA3')

Bax:

(F 5' GTCCCGCCTCTTCAC CTT TCAG 3'; R 5'GATTCTGGTGTTTCCCCGTTGG 3')

Caspase 3:

(F 5'GGACAGCAGTTACAAAATGGATTA 3'; R 5' CGGCA GGCC TGAA TGAT GAAG 3')

Periostin:

(F 5' FACGGAGCTCAGGGCTGAAGATG 3'; R 5'GTTT GGGCCC TGA TCCCGAC 3')

Fibronectin 1 (Fn):

(F 5' CGAAGAGCCCTTACAGTTCCA 3', R 5' ATCTGTA GGCTGGT TCAGGC 3')

Actin Alpha 2 (Acta2):

(F 5'AGATCAAG ATCATTGCCCCTCC 3', R 5' TTGTGTGCTAGAGGCAGAGC 3')

miR 133-a: (MIMAT0000427: 5' UUUGGUCCCC UUCAACCAGCUG).

Legends

Figure S1. **Characterization of EV.** A. Nanoparticle tracking analysis of EV-CPC: The preparation detected a poly-disperse population of particles ranging primarily from approximately 50 to 550 nm, with predominantly small particles. B. Representative cryo-TEM images of phosphatidylserine+ EVs (labeled by Anx5-goldNps) (1,2), and CD81+ EVs (3,4). C. Western Blot of lysed EV-CPC for HSC70. D. Western Blot of EV-CPC for CD63 and CD9. E. Western Blot of EV-CPC for Calnexin (CNX). MW: Molecular Weight, CPC: Cardiovascular Progenitor cells, EV-CPC : Extracellular vesicles from iPS cell derived cardiovascular progenitor cells), Nps : Nanoparticles.

Figure S2. Effects of EV-CPC on echocardiographically-measured left ventricular volumes in MerCreMer/ZEG mice. A. Left ventricular end systolic volume and left ventricular end diastolic volume expressed at baseline (pre-transplantation, 3 weeks following MI) and at the end of study or B. As percent changes from baseline. Each group comprised 15 mice.

Figure S3. **Effects of EV-CPC on fibrosis in MerCreMer/ZEG mice.** A. Fibrotic area measured using Masson trichrome staining. B. Interstitial fibrosis measured using red Sirius staining, a fixed area is delimited in peri-infarcted zones and quantified. $*p \leq 0.05$. C. Expression levels of fibrotic genes quantified by real time quantitative PCR (qRT-PCR) in 5 EV-treated and 6 control MerCreMer/ZEG hearts, respectively.

Figure S4. **Expression levels of fibrotic, apoptotic and autophagic genes in C7BL6J mice.** Genes were quantified by real time quantitative PCR (qRT-PCR) in 7 and 6-7 EV-treated and control C7BL6J mice hearts, respectively 3 days post EV treatment and 5 days post MI. $*p = 0.03$, $**p = 0.005$.

Figure S5. Effects of EV-CPC on echocardiographically-measured left ventricular volumes in C57BL/6J mice. A. Left ventricular end systolic volume and left ventricular end diastolic volume expressed at baseline (pre-transplantation, 3 weeks following MI) and at the end of study or B. As percent changes from baseline. Each group comprised 6 mice.

References

1. El Harane N, Kervadec A, Bellamy V, Pidial L, Neametalla HJ, Perier MC, et al. Acellular therapeutic approach for heart failure: in vitro production of extracellular vesicles from human cardiovascular progenitors. Eur Heart J. 2018; 39: 1835–47.

2. Lima Correa B, El Harane N, Gomez I, et al. Extracellular vesicles from human cardiovascular progenitors trigger a reparative immune response in infarcted hearts. Cardiovasc Res. 2021; 117: 292–307.

3. Théry C, Witwer KW, Aikawa E, Alcaraz MJ, Anderson JD, Andriantsitohaina R, et al. Minimal information for studies of extracellular vesicles 2018 (MISEV2018): a position statement of the International Society for Extracellular Vesicles and update of the MISEV2014 guidelines. J Extracell Vesicles. 2018; 7: 1535750.

4. Arraud N, Linares R, Tan S, Gounou C, Pasquet JM, Mornet S, et al. Extracellular vesicles from blood plasma: determination of their morphology, size, phenotype and concentration. J Thromb Haemost. 2014; 12: 614–27.

5. Chan SS-K, Shueh Y-Z, Bustamante N, Tsai SJ, Wu HL, Chen JH, et al. Genetic fate-mapping for studying adult cardiomyocyte replenishment after myocardial injury. Methods Mol Biol. 2010; 660: 201–11.

6. Toeg HD, Tiwari-Pandey R, Seymour R, Ahmadi A, Crowe S, Vulesevic B, et al. Injectable small intestine submucosal extracellular matrix in an acute myocardial infarction model. Ann Thorac Surg. 2013; 96: 1686–94.

7. Kervadec A, Bellamy V, El Harane N, Arakélian L, Vanneaux V, Cacciapuoti I, et al. Cardiovascular progenitor-derived extracellular vesicles recapitulate the beneficial effects of their parent cells in the treatment of chronic heart failure. J Heart Lung Transplant. 2016; 35: 795–807.

Figure S1

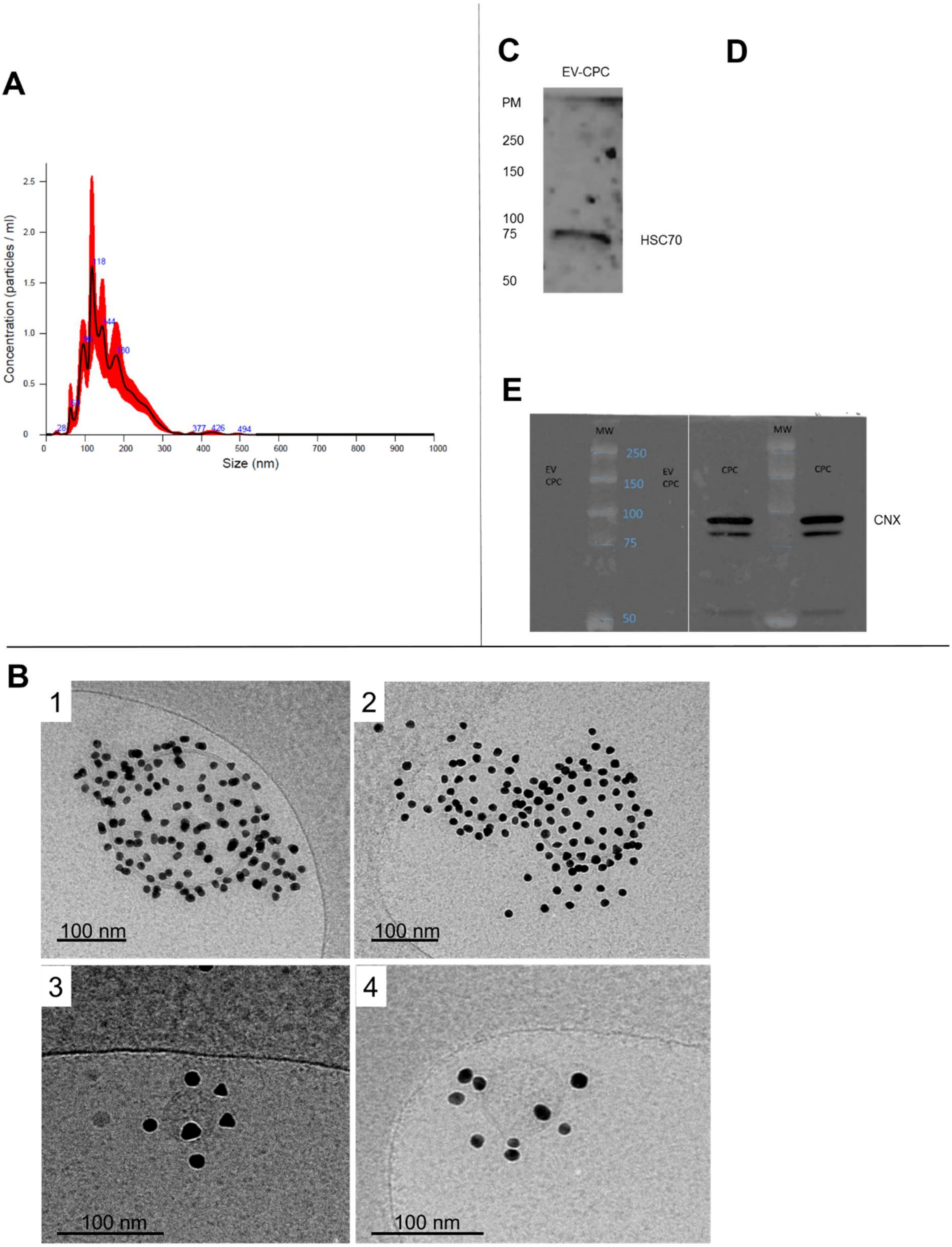
A
Concentration (particles / ml)
Size (nm)
2.5
2.0
1.5
1.0
0.5
0
100
200
300
400
500
600
700
800
900
1000
118
144
180
28
377
426
494
B
1
2
3
4
100 nm
100 nm
100 nm
100 nm
C
EV-CPC
PM
250
150
100
75
50
HSC70
D
E
MW
250
150
100
75
50
EV CPC
EV CPC
MW
CPC
CPC
CNX

Figure S2

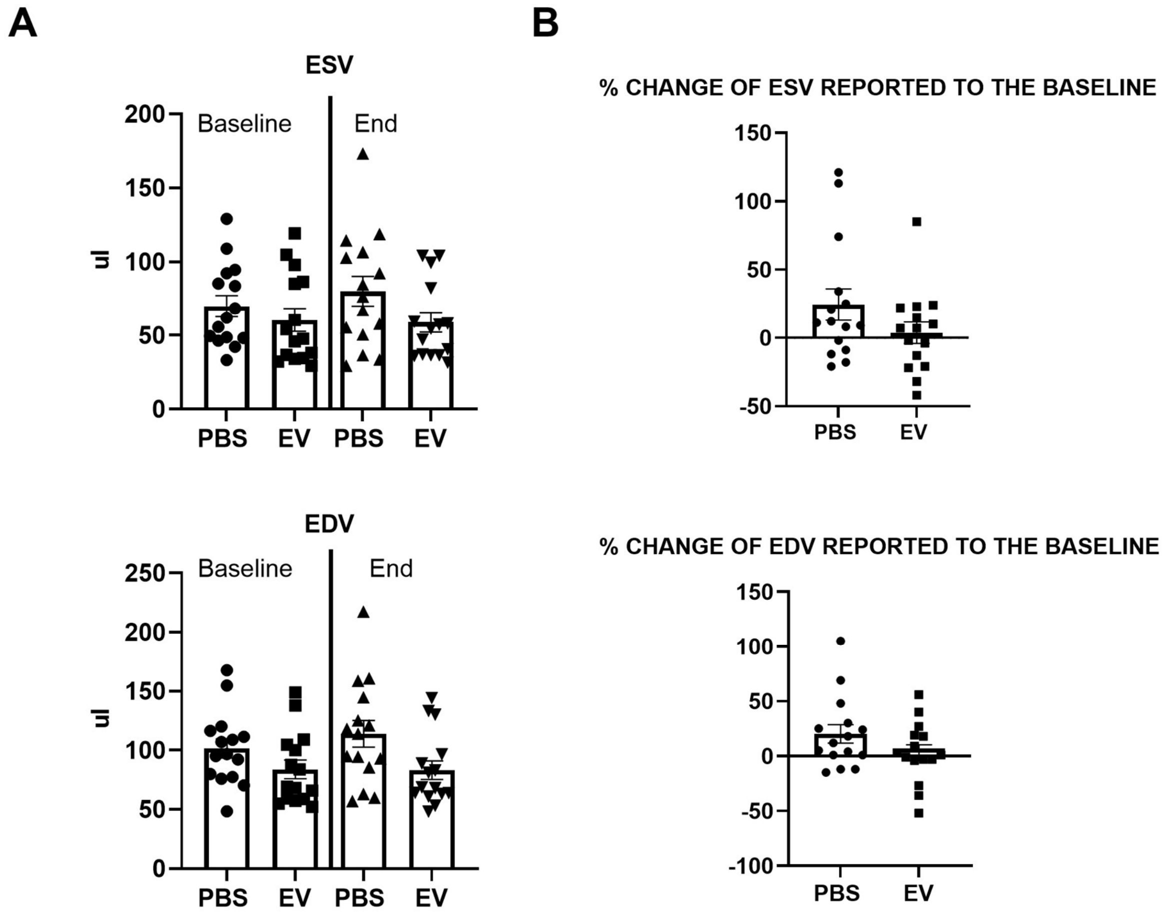
A
ESV
Baseline
End
200
150
100
50
0
ul
PBS
EV
PBS
EV
EDV
Baseline
End
250
200
150
100
50
0
ul
PBS
EV
PBS
EV
B
% CHANGE OF ESV REPORTED TO THE BASELINE
150
100
50
0
-50
PBS
EV
% CHANGE OF EDV REPORTED TO THE BASELINE
150
100
50
0
-50
-100
PBS
EV

Figure S3

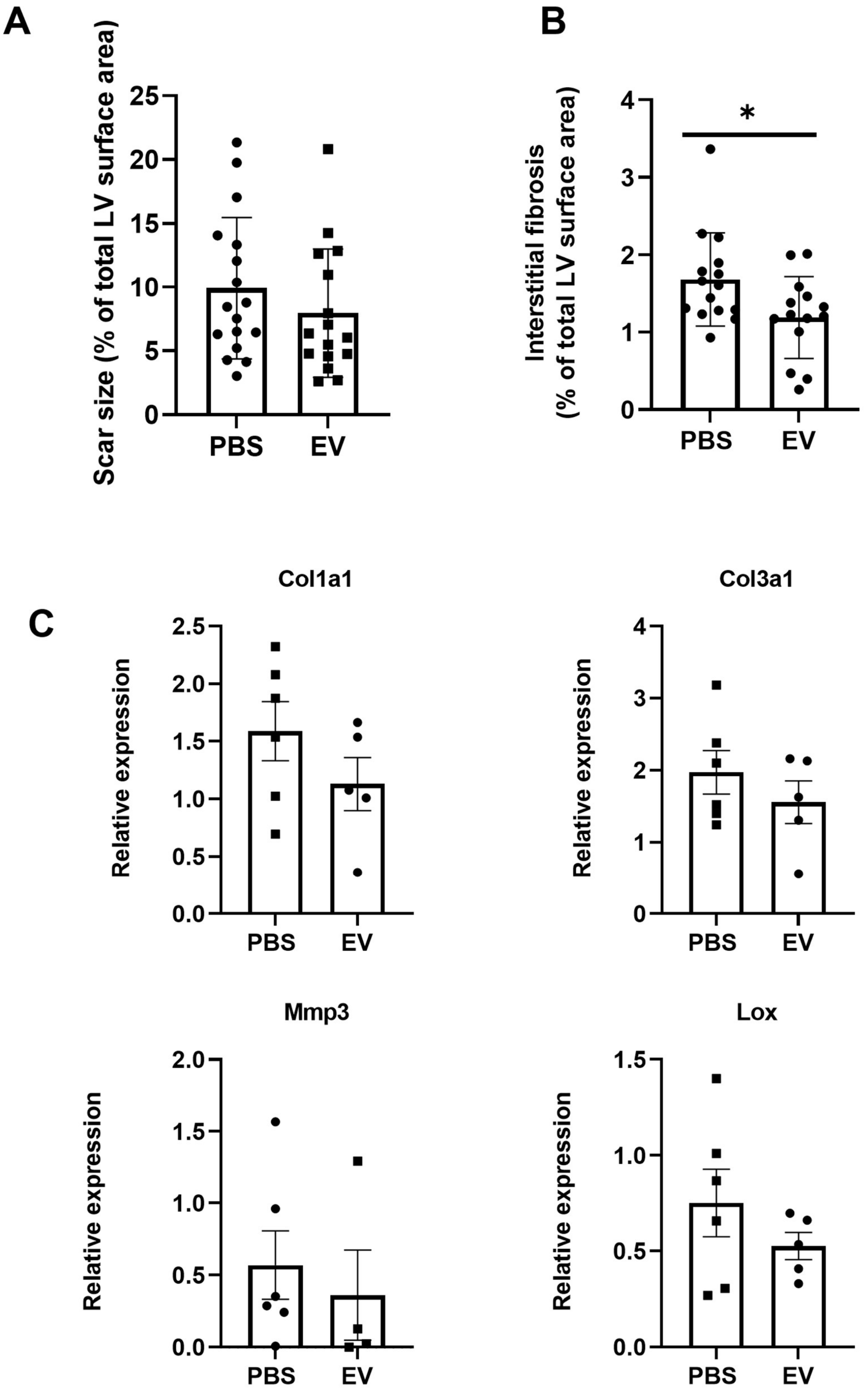
A
Scar size (% of total LV surface area)
25
20
15
10
5
0
PBS
EV
B
Interstitial fibrosis
(% of total LV surface area)
4
3
2
1
0
*
PBS
EV
C
Col1a1
Relative expression
2.5
2.0
1.5
1.0
0.5
0.0
PBS
EV
Col3a1
Relative expression
4
3
2
1
0
PBS
EV
Mmp3
Relative expression
2.0
1.5
1.0
0.5
0.0
PBS
EV
Lox
Relative expression
1.5
1.0
0.5
0.0
PBS
EV

Figure S4

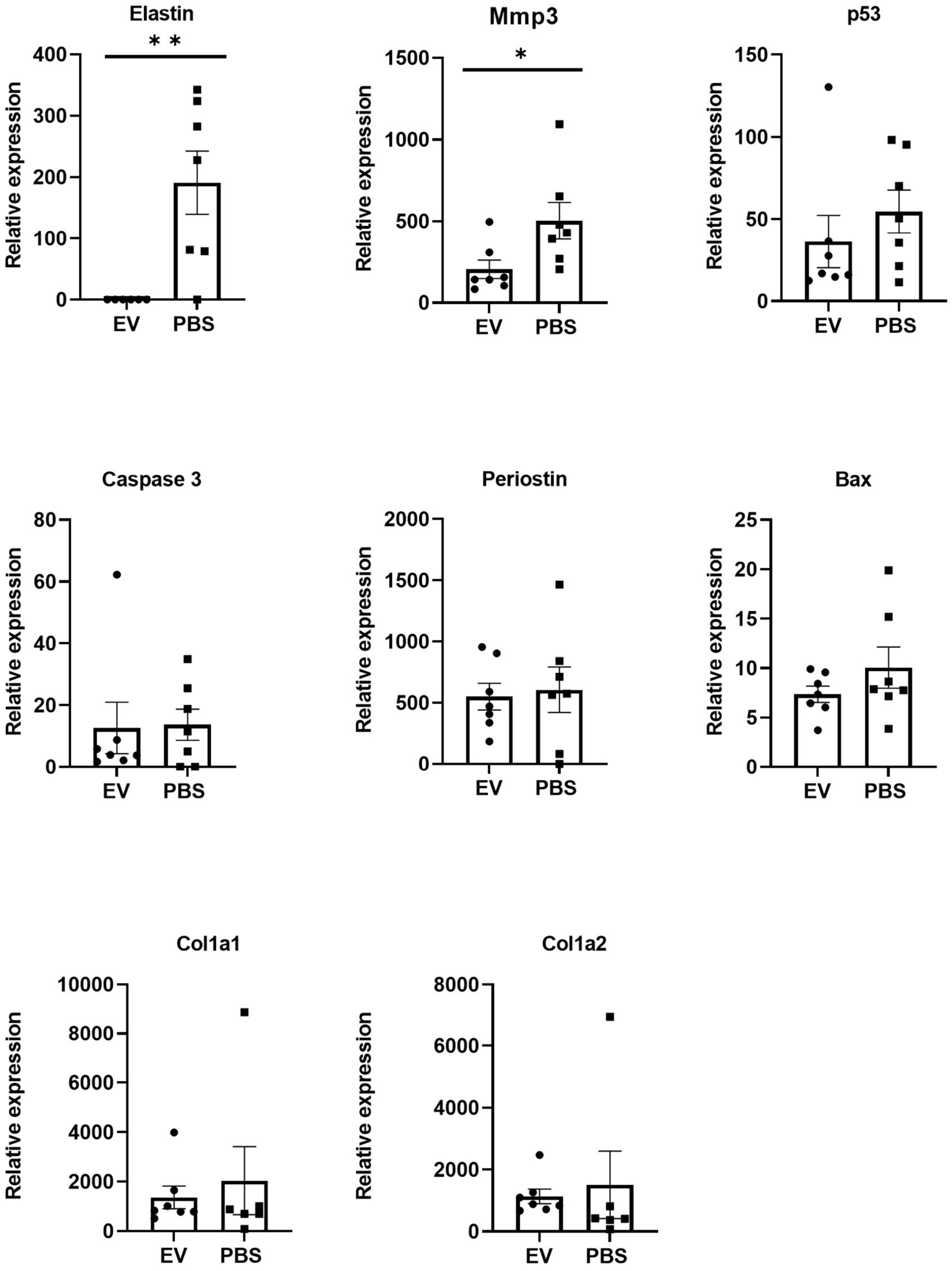
Elastin
**
Relative expression
400
300
200
100
0
EV
PBS
Mmp3
*
1500
1000
500
0
p53
150
100
50
Caspase 3
80
60
40
20
Periostin
2000
1500
1000
500
Bax
25
20
15
10
5
Col1a1
10000
8000
6000
4000
2000
Col1a2
8000
6000
4000
2000

Figure S5

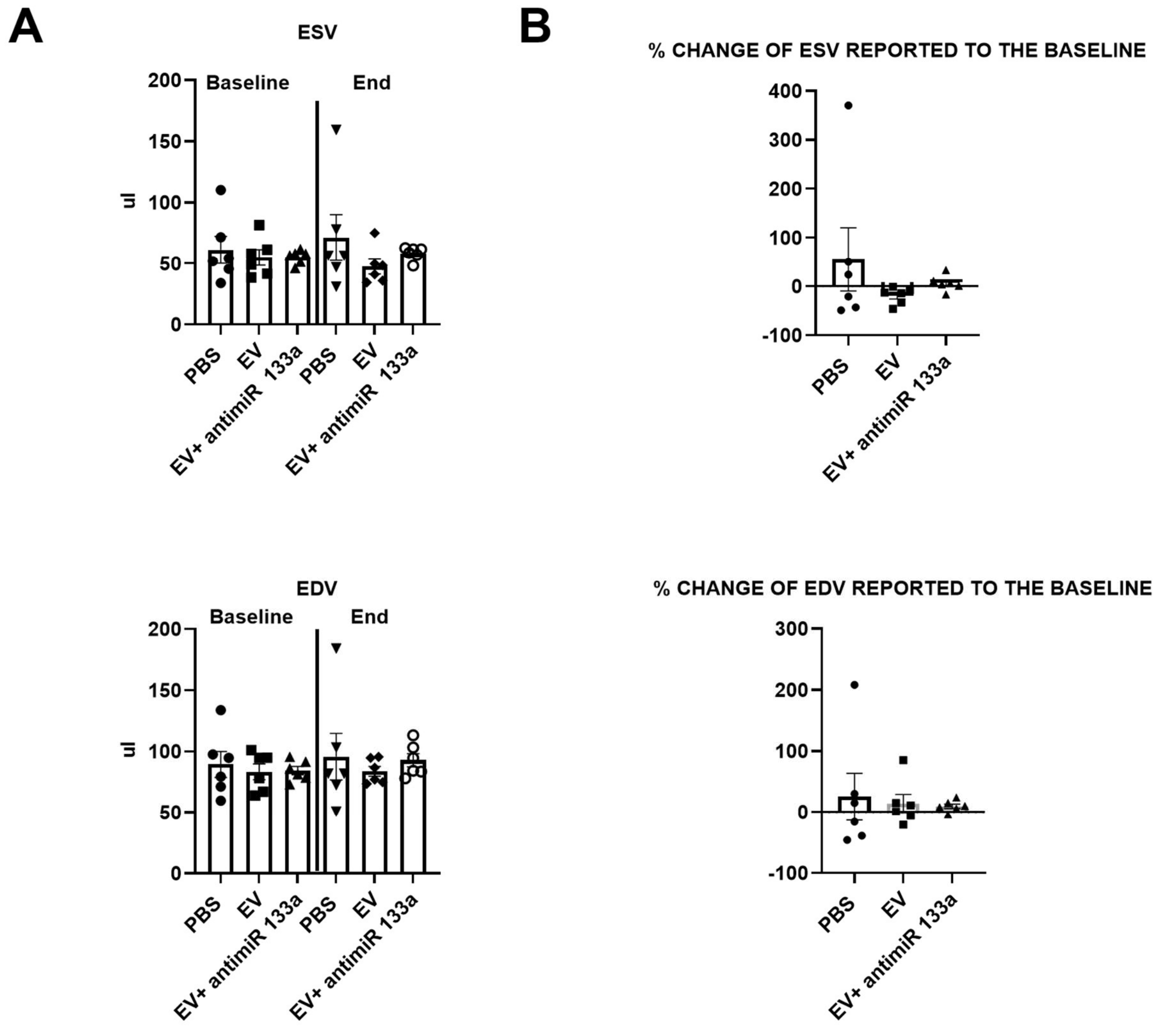
A
ESV
Baseline
End
ul
200
150
100
50
0
PBS
EV
EV+ antimiR 133a
EDV
B
% CHANGE OF ESV REPORTED TO THE BASELINE
400
300
200
100
-100
% CHANGE OF EDV REPORTED TO THE BASELINE
